# Chemical Compatibility of Li_1.3_Al_0.3_Ti_1.7_(PO_4_)_3_ Solid-State Electrolyte Co-Sintered with Li_4_Ti_5_O_12_ Anode for Multilayer Ceramic Lithium Batteries

**DOI:** 10.3390/ma18040851

**Published:** 2025-02-15

**Authors:** Jiangtao Li, Mingsheng Ma, Ya Mao, Faqiang Zhang, Jingjing Feng, Yingchun Lyu, Tu Lan, Yongxiang Li, Zhifu Liu

**Affiliations:** 1 State Key Laboratory of High Performance Ceramics and Superfine Microstructure, Shanghai Institute of Ceramics, Chinese Academy of Sciences, Shanghai 201899, China; lijiangtao22@mails.ucas.ac.cn (J.L.);; 2 Center of Materials Science and Optoelectronics Engineering, University of Chinese Academy of Sciences, Beijing 100049, China; 3 State Key Laboratory of Space Power Sources, Shanghai Institute of Space Power-Sources, Shanghai 200245, China; 4 Materials Genome Institute, Shanghai University, Shanghai 200444, China; 5 School of Engineering, RMIT University, Melbourne 3000, Australia

**Keywords:** MLCBs, LATP, LTO, composite electrodes, co-sintering

## Abstract

Multilayer ceramic lithium batteries (MLCBs) are regarded as a new type of oxide-based all-solid-state microbattery for integrated circuits and various wearable devices. The chemical compatibility between the solid electrolyte and electrode active materials during the high-temperature co-sintering process is crucial for determining the structural stability and cycling performance of MLCBs. This study focuses on the typical MLCB composite electrodes composed of the NASICON-type Li_1.3_Al_0.3_Ti_1.7_(PO_4_)_3_ (LATP) solid electrolyte and the spinel-type Li_4_Ti_5_O_12_ (LTO) anode material. The thermal behavior, phase structure, morphological evolution, and elemental chemical states of these composite electrodes were systematically investigated over a co-sintering temperature range of 400–900 °C. The results indicate that the reactivity between LATP and LTO during co-sintering is primarily driven by the diffusion of Li from the LTO anode, leading to the formation of TiO_2_, Li_3_PO_4_, and LiTiOPO_4_. Furthermore, the co-sintered LATP-LTO multilayer composites reveal that the generation of Li_3_PO_4_ at the LATP/LTO interface facilitates their co-sintering integration at 800–900 °C, which is essential for the successful fabrication of MLCBs. These findings provide direct evidence and valuable references for the structural and performance optimization of MLCBs in the future.

## 1. Introduction

The rapid advancement of micro-nano electronics and the rise of Intelligence of Things (IoT) technologies have significantly accelerated the development of miniature electronic devices and integrated microsystems, such as smart wearables, healthcare devices, and micro/nanorobots. These innovations are central to the rapidly expanding IoT ecosystem, covering various fields including smart homes, industrial automation, smart cities, and mobile healthcare. Meanwhile, the integrated circuits and functional modules in these IoT devices urgently require new micropower solutions that synergize miniaturization, high integration, safety, and durability [1,2,3,4]. Currently, micro lithium-ion batteries (MBs) have emerged as the preferred power sources for IoT devices due to their superior performance, including high energy density, long cycle life, and low self-discharge rate [5,6].

Conventional MBs primarily consist of button cells and all-solid-state thin-film batteries [7]. The button cells are widely used in IoT devices due to their mature manufacturing processes and cost advantages. However, they face issues such as electrolyte leakage and thermal runaway, and there are size constraints, limiting their ability to meet the demands of a micropower source in IoT device [8,9]. All-solid-state thin-film batteries replace organic liquid electrolytes with solid-state electrolytes, fundamentally eliminating flammability risks. For example, thin-film lithium batteries using the LiPON solid electrolyte, which is deposited via radio-frequency magnetron sputtering, offer a cycle life exceeding 10,000 cycles and a capacity retention rate above 90% [10]. It is worthwhile to mention that the high production costs and low manufacturing efficiency restrict their large-scale application [11,12].

A promising solution to these challenges is the development of multilayer ceramic lithium batteries (MLCBs), which employ multilayer ceramic technology similar to that used in multilayer ceramic capacitors (MLCCs), achieving co-sintering integration of the solid-state electrolyte, electrodes, and metal current collectors [13]. As a new type of all-solid-state micro lithium battery, the dense ceramic structure in MLCBs enhances interfacial ion transport and achieves high reliability and safety. Additionally, the MLCBs are compatible with surface-mount technology (SMT), allowing for their direct integration into the printed circuit boards (PCBs) or sensor modules, facilitating seamless miniaturization in smart systems. Therefore, MLCBs not only fully meet the demands of IoT devices for miniaturized, highly integrated, safe, and durable micropower sources but also possess advantages such as low-cost and high-efficiency mass production, similar to MLCCs [14,15].

Unlike the mostly reported all-solid-state batteries (ASSBs) that utilize lithium metal anodes [16], MLCBs employ ceramic materials for both the electrolytes and electrodes and are fabricated through a single-step co-sintering process. During the co-sintering of MLCBs, the solid-state electrolytes often undergo chemical reactions with electrode active materials, forming high-resistance interfacial phases that degrade the battery’s structure and performance [17,18]. Therefore, investigating the chemical compatibility between solid-state electrolytes and electrode active materials during co-sintering is essential for optimizing the design and fabrication of MLCBs. The previous studies in ASSBs mainly focus on the chemical compatibility between oxide-based solid-state electrolytes and different cathode active materials, such as garnet-type or NASICON-type solid-state electrolytes with layered LiMO_2_ cathodes, spinel-type LiM_2_O_4_ cathodes, and olivine-type LiMPO_4_ cathodes (M = transition metals), as these systems typically use lithium metal anodes [19,20,21,22]. It is worth noting that there is limited research on the chemical compatibility between solid-state electrolytes and anode active materials. However, understanding the chemical compatibility of solid electrolytes with both cathode and anode materials during co-sintering is equally important in MLCBs.

The most widely studied lithium-based oxide solid electrolytes are primarily classified into three categories: NASICON-type Li_1+*x*_Al*_x_*Ti_2−*x*_(PO_4_)_3_ (LATP), garnet-type Li_7_La_3_Zr_2_O_12_ (LLZO), and perovskite-type Li_3*x*_La_2/3−*x*_TiO_3_ (LLTO) [23]. Among these, the NASICON-type LATP solid electrolyte, which was developed from the Na^+^ super ionic conductor (NASICON) NaM_2_(PO_4_)_3_ (M = Ge, Ti, and Zr) with the Na substituted by Li [24], achieves a high ionic conductivity greater than 1 × 10^−3^ S/cm at room temperature, along with excellent air and thermal stability. It also has a low sintering temperature and uses cost-effective raw materials, making it an ideal choice for oxide solid electrolytes in MLCBs [25]. The spinel-type oxide Li_4_Ti_5_O_12_ (LTO) is the most commonly used oxide-based anode material. LTO offers exceptional cycling stability and rate performance, primarily due to its “zero-strain” properties during lithium-ion insertion and extraction, as well as its three-dimensional (3D) lithium-ion transport pathways. These properties are crucial for the performance of MLCBs [26,27]. To investigate the feasibility of applying LATP and LTO in MLCBs, there is an urgent need to conduct a systematic study of the chemical compatibility between LATP and LTO during co-sintering.

The rigid solid–solid contact between the solid electrolyte and the electrodes in ASSBs leads to a high interfacial resistance. In order to enhance the ionic conductivity and improve interfacial contact, a common approach is to incorporate solid electrolytes into the electrodes to form composite electrodes [28,29,30]. Therefore, composite electrodes were prepared by mixing LATP and LTO powders in this work, and various characterization techniques were employed to investigate the thermal behavior, phase structure, morphological evolution, and elemental chemical states of the LATP-LTO composite electrodes at different co-sintering temperatures. Furthermore, LATP-LTO multilayer composite sheets were fabricated to study the multilayer co-sintering characteristics of LATP and LTO. The findings aim to provide theoretical insights into the application of LATP and LTO in MLCBs.

## 2. Materials and Methods

### 2.1. Preparation of LATP-LTO Composite Electrode Pellets

The Li_1.3_Al_0.3_Ti_1.7_(PO_4_)_3_ (LATP) solid-state electrolyte (RM-EGY-01N-0000, Ganfeng LiEnergy, Xinyu, China) and the Li_4_Ti_5_O_12_ (LTO) anode material (MS-LTO-2, Shenzhen Kejing Star Technology Company, Shenzhen, China) were purchased and used as received. The LATP-LTO composite electrode pellets (hereinafter referred to as PT pellets) were prepared according to the method reported by Miara et al. [19]. Equal volumes of LATP and LTO powders were dispersed in anhydrous ethanol containing zirconia balls and ball-milled for 6 h. After drying and sieving, the PT mixed powder was pressed into 10 mm diameter green pellets under a uniaxial pressure of 300 MPa. The green pellets were subsequently co-sintered in air at temperatures ranging from 400 °C to 900 °C for 3 h, with a heating rate of 5 °C/min. Single LATP and LTO pellets were also prepared using the same procedure and sintered at temperatures between 800 °C and 1000 °C for 3 h, respectively.

### 2.2. Preparation of LATP-LTO Multilayer Composite Sheets

To prepare LATP-LTO multilayer composite sheets (hereinafter referred to as PT sheets), the LATP and LTO green tapes were first fabricated by the tape-casting method. The obtained green tapes were cut into 200 mm × 160 mm sheets and alternately stacked to form PT green sheets. Subsequently, they were subjected to hot isostatic pressing at 70 °C and then cut into 5.7 mm × 6.3 mm green sheets. Finally, the green sheets were heated in air at a rate of 1 °C/min to 450 °C and held for 2 h to fully remove organic binders, followed by heating at 5 °C/min to 800 °C and 900 °C for 30 min to complete the co-sintering process.

### 2.3. Sample Characterization

The LATP, LTO, and their mixed powders were first characterized using thermogravimetric-differential scanning calorimetry (TG-DSC, Mettler TGA/DSC1, Greifensee, Switzerland) under N_2_/O_2_ atmospheres. The samples were heated from room temperature to 1100 °C at a rate of 10 °C/min and then cooled to room temperature. The co-sintered PT pellets were ground into fine powders and subjected to X-ray diffraction (XRD) analysis using a high-resolution powder X-ray diffractometer (Bruker D8 Advance, Ettlingen, Germany) with Cu Kα radiation. The scan range 2θ was 10–80° with a step size of 0.02° and a dwell time of 0.3 s per step. The morphologies of the cross-sections of the PT pellets co-sintered at various temperatures, as well as LATP and LTO powders, were examined using a high-resolution field emission scanning electron microscope (FESEM, Thermo Scientific Verios G4, Waltham, MA, USA). The elemental chemical states in PT pellets co-sintered at different temperatures were investigated using X-ray photoelectron spectroscopy (XPS, Thermo Scientific, K-Alpha+, Waltham, MA, USA). For PT pellets co-sintered at 800 °C, thin lamella for transmission electron microscopy (TEM) were prepared using a focused ion beam–electron beam dual-beam system (FEI Versa 3D, Hillsboro, OR, USA). The elemental distributions and phase structures were analyzed using a field emission TEM (FEI TECNAI G2 F20, Hillsboro, OR, USA) equipped with energy-dispersive spectroscopy (EDS). The cross-sections of co-sintered PT sheets were mechanically ground and polished. Morphology analysis and EDS characterization of these cross-sections were conducted using an ultra-high-resolution FESEM (TESCAN CLARA, Brno, Czech Republic).

## 3. Results and Discussion

**Figure 1:**
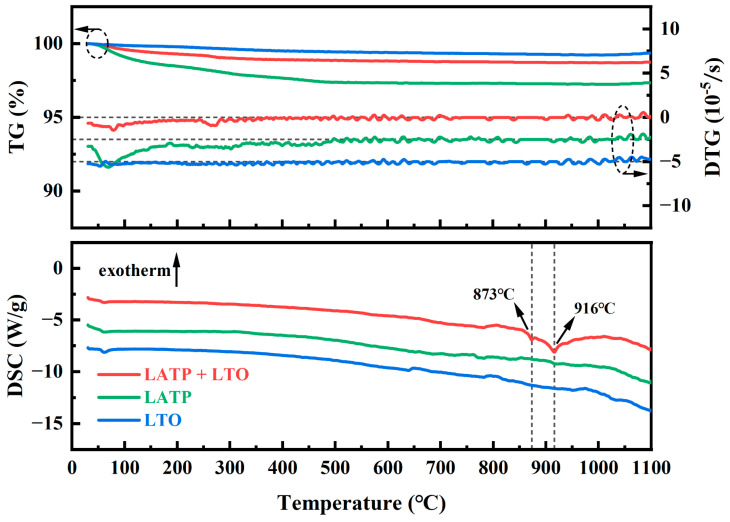
TG-DSC curves of LATP, LTO, and their mixtures. DTG signal offset: 2.5 unit (for LATP); 5 unit (for LTO). DSC signal offset: 5 unit (for LATP); 10 unit (for LTO).

To investigate the thermal stability of LATP, LTO, and their mixed powders at high temperatures, we initially studied their thermal behavior over a temperature range from room temperature to 1100 °C using simultaneous thermal analysis, as shown in Figure 1. After the release of surface-adsorbed H₂O and CO₂, both the LATP and LTO powders exhibit similar thermal behavior, with nearly constant TG signals and no significant endothermic or exothermic peaks in their DSC curves. These results demonstrate that LATP and LTO powders possess good thermal stability up to 1100 °C. In contrast, the LATP-LTO mixed powders display distinct thermal behavior, characterized by two endothermic peaks at approximately 873 °C and 916 °C. These peaks are not observed in the TG-DSC curves of pure LATP and LTO powders, suggesting that they are likely related to the reaction between LATP and LTO at high temperatures. Additionally, it has been reported that LATP mixed in equal volumes with high-voltage spinel cathode LiM_2_O_4_ (M = Fe, Co, and Ni combined with Mn) exhibits 5–6 wt% weight loss at high temperatures due to oxygen release [19]. However, the LATP-LTO mixed powders exhibit negligible weight loss at high temperatures, which may be attributed to the strong covalent bonds formed between Ti and O in LTO [31,32].

**Figure 2:**
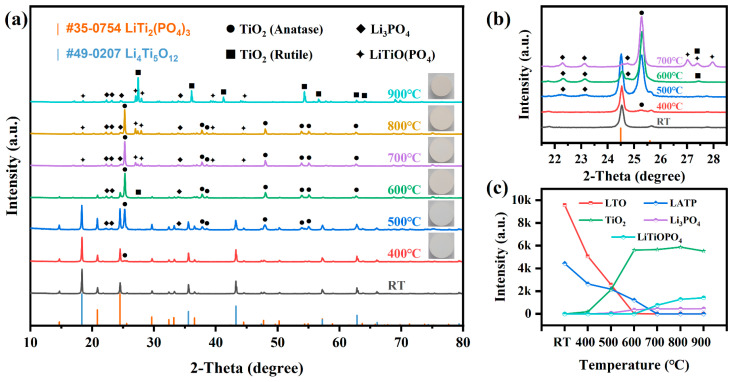
XRD pattern of (**a**) LATP-LTO composite electrode pellets co-sintered at 400–900 °C and (**b**) enlarged view of the 2-Theta regions 21.5–28.5°. (**c**) Evolution of phase structures and variations in the diffraction intensities of the strongest peaks corresponding to different phases in LATP-LTO composite electrode pellets after co-sintering at various temperatures.

Figure 2 shows the XRD patterns and phase evolution of PT pellets co-sintered in air at 400–900 °C for 3 h. For comparison, Figures S1 and S2 show the XRD patterns of LATP and LTO pellets sintered individually at 800–1000 °C for 3 h. In LATP, the primary phase structure of LATP remained stable, although a small amount of AlPO_4_ phase was observed, attributed to trace impurities in the raw materials. Additionally, a minor LiTiOPO_4_ phase was detected at 1000 °C. Meanwhile, the phase structure of LTO remained unchanged across the sintering temperature range of 800–1000 °C. These results confirm the excellent phase stability of both LATP and LTO below 1000 °C.

For PT pellets, as the co-sintering temperature increased from 400 °C to 600 °C, the intensity of LATP and LTO diffraction peaks gradually decreased. The XRD patterns of PT pellets began to show major changes at 500 °C, with the formation of two new phases, TiO_2_ and Li_3_PO_4_. At 600 °C, TiO_2_ became the main crystalline phase. When the co-sintering temperature was further increased to 700 °C, a new secondary phase which can be identified as LiTiOPO_4_ appeared. This is likely related to the increased content of Li_3_PO_4_, which causes the decomposition of LATP into LiTiOPO_4_ and AlPO_4_ [33]. At this point, no diffraction peaks corresponding to LATP or LTO were observed, and the phase composition of PT pellets began to stabilize, mainly consisting of anatase TiO_2_, Li_3_PO_4_, and LiTiOPO_4_. Finally, after co-sintering at 900 °C, the resulting TiO_2_ phase was primarily rutile, owing to the higher thermodynamic stability of rutile TiO_2_ compared to anatase TiO_2_ at 900 °C [34,35]. Additionally, no secondary phases containing an Al element were observed after co-sintering, implying that the Al element was contained in amorphous phases, which are undetectable due to the limitations of XRD.

**Figure 3:**
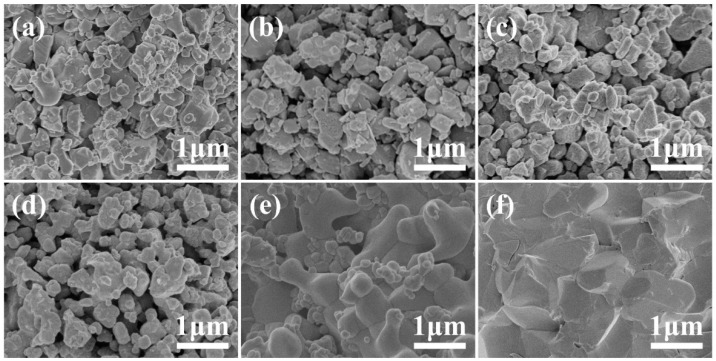
FESEM images of the cross-sectional morphology of LATP-LTO composite electrode pellets co-sintered at different temperatures: (**a**) 400 °C, (**b**) 500 °C, (**c**) 600 °C, (**d**) 700 °C, (**e**) 800 °C, and (**f**) 900 °C.

As the co-sintering temperature increases, the evolution of the phase structure is accompanied by significant changes in morphology. Figure 3 shows the evolution of the cross-sectional morphology of PT pellets after co-sintering at 400–900 °C. For comparison, Figure S3 shows the original morphology of LATP and LTO powders, as well as the cross-sectional images of PT green pellets before co-sintering. When the co-sintering temperature increased from 400 °C to 600 °C, the surface morphology of LATP and LTO particles gradually changed from smooth to rough. At 600 °C, the morphology of LATP and LTO particles in the PT pellets transformed into aggregates of fine secondary particles. As the co-sintering temperature was further increased to 700 °C, the secondary particles became larger and showed a tendency to sinter and grow. When the co-sintering temperature reached 800 °C, the PT pellets exhibited good densification, accompanied by some smaller secondary particles. The densification phenomenon was associated with the Li_3_PO_4_ phase observed in the previous XRD phase evolution analysis. Li_3_PO_4_ is a common sintering aid with excellent chemical and thermal stability. Its melting point is approximately 837 °C, and it can promote the sintering of LATP at around 700 °C [33,36]. Finally, after co-sintering at 900 °C, the PT pellets showed significant grain growth and achieved full densification.

**Figure 4:**
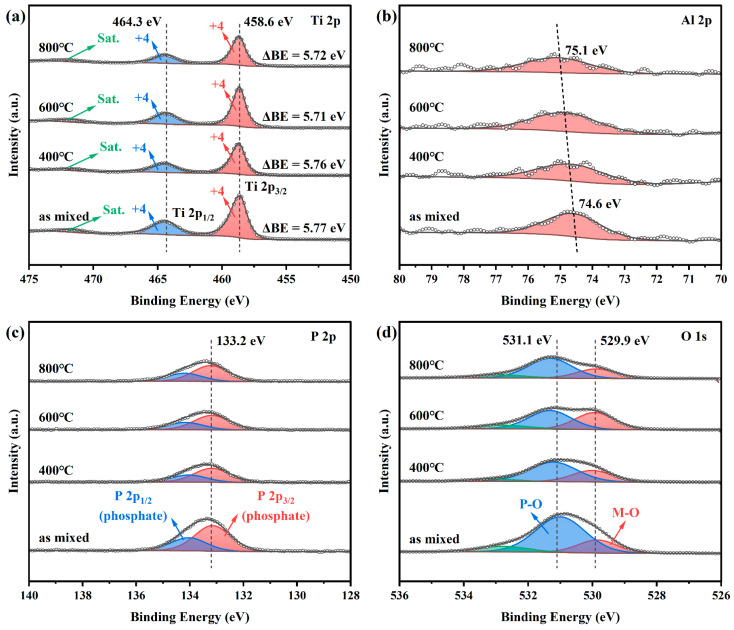
XPS high-resolution spectra of LATP-LTO composite electrode pellets co-sintered at different temperatures: (**a**) Ti 2p, (**b**) Al 2p, (**c**) P 2p, and (**d**) O 1s.

Since chemical reactions during co-sintering would lead to changes in the chemical states of elements, XPS was employed to investigate the potential changes in chemical states. Figure 4 shows the high-resolution XPS spectra of Ti 2p, Al 2p, P 2p, and O 1s in PT pellets co-sintered at different temperatures, and all binding energies have been calibrated using the adventitious carbon C 1s peak at 284.8 eV. The Ti 2p spectrum consistently exhibits two distinct peaks near 458.6 eV (Ti 2p_3/2_) and 464.3 eV (Ti 2p_1/2_) with a spin-orbit splitting energy of ~5.7 eV, along with a satellite peak around 472 eV. These features are typical characteristic of Ti^4+^, suggesting that Ti remains in the Ti^4+^ oxidation state throughout the co-sintering process [37]. For Al 2p, the peak is observed at approximately 74.6 eV before co-sintering. As the co-sintering temperature increases, the Al 2p peak gradually shifts to higher binding energies, reaching 75.1 eV after co-sintering at 800 °C. This shift is likely due to the formation of AlPO_4_ [38,39]. In contrast, the P 2p peak shows no significant change during co-sintering, and the P 2p_3/2_ peak is consistently found around 133.2 eV, indicating that P remains in the form of phosphates, which suggests the stability of the phosphate structure during the co-sintering process [40]. Additionally, the O 1s spectra primarily shows two peaks: the M-O peak near 529.9 eV and the P-O peak near 531.1 eV [40,41]. After co-sintering, the intensity of the M-O peak is enhanced, indicating an increase in the concentration of metal oxides, which relates to the formation of TiO_2_ [42]. Meanwhile, the P-O peak exhibits a slight shift towards higher binding energies (<0.2 eV) after co-sintering at 600 °C and 800 °C, which is likely associated with the decomposition of LATP to form Li_3_PO_4_ as the increased concentration of Li^+^ around the PO₄ tetrahedra leads to the rise in the O 1s binding energy [43,44]. Therefore, based on the above analysis, it is clear that the valence states of elements in PT pellets show no significant changes before and after co-sintering, which is consistent with the TG-DSC results and the XRD phase evolution analysis of PT pellets.

**Figure 5:**
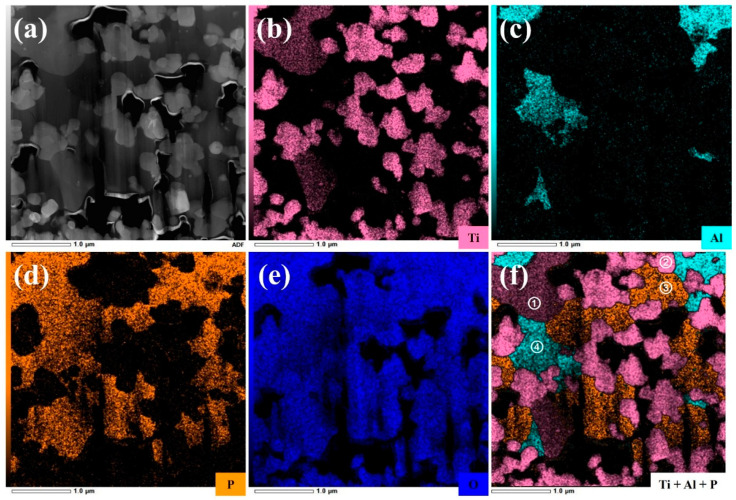
TEM-EDS mapping of LATP-LTO composite electrode pellet co-sintered at 800 °C: (**a**) TEM image; (**b**–**f**) corresponding to Ti, Al, P, O, and their EDS overlays.

**Figure 6:**
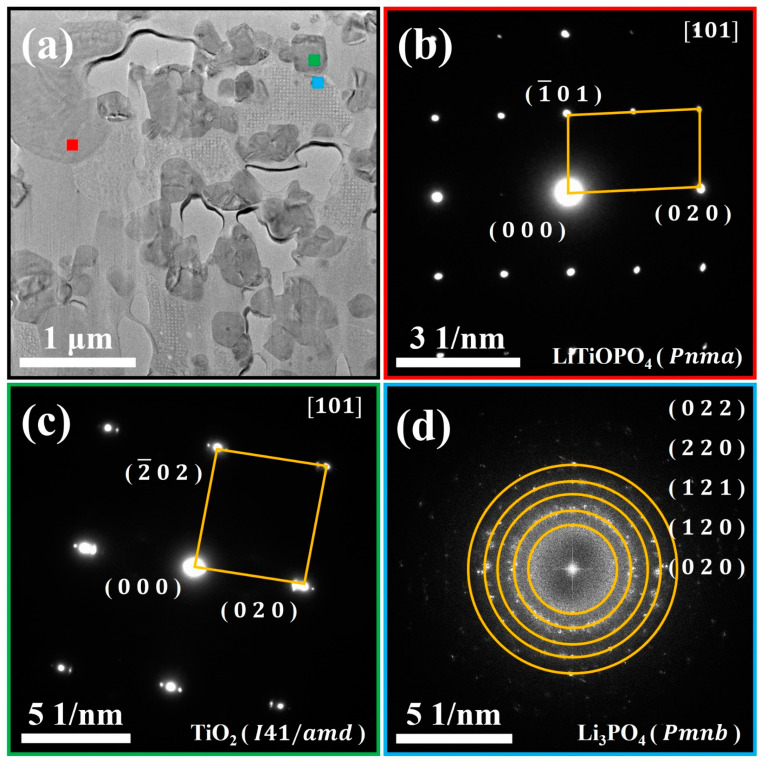
(**a**) TEM image of LATP-LTO composite electrode pellet co-sintered at 800 °C, (**b**,**c**) SAED pattern corresponding to the red and green square in (**a**), and (**d**) FFT corresponding to the blue square in (**a**).

In order to have a better understanding of the phase composition and elemental distribution of PT pellets after co-sintering, further revealing their reaction mechanism during the co-sintering process, the TEM and EDS analyses were carried out. Figure 5 shows the TEM images of the PT pellets co-sintered at 800 °C and the EDS mappings of the Ti, Al, P, and O elements, along with their overlays. Based on the distribution of different elements, four distinct regions are classified and labeled as region ① to ④ in Figure 5f:

(1) Region ①: this region predominantly consists of Ti, P, and O elements. Combined with the SAED pattern shown in Figure 6b, it can be confirmed as the LiTiOPO_4_ phase;
(2) Region ②: this region predominantly consists of Ti and O elements. The SAED results in Figure 6c confirm that it corresponds to anatase TiO_2_;
(3) Region ③: this region predominantly consists of P and O elements. As shown in Figure 6d and Figure S4b,d it comprises polycrystalline Li_3_PO_4_ and an amorphous phase;
(4) Region ④: this region predominantly contains Al, P, and O elements, likely corresponding to the formation of AlPO_4_. Figure S4b,c reveal that this region is amorphous, which explains the absence of Al-containing secondary phases in the XRD analysis.

Therefore, the above analysis further indicates that the reaction products of the PT pellets after co-sintering are mainly composed of TiO_2_, LiTiOPO_4_, Li_3_PO_4_, and amorphous Al-P-O compounds. In addition, an interesting phenomenon is observed: the Ti and Al elements are found to be separated, while LiTiOPO_4_ is primarily derived from the decomposition of LATP. This suggests that the reaction between LATP and LTO during high-temperature co-sintering is an in situ decomposition process, which can be explained by the diffusion of Li. According to previous studies [27,45,46], the Li element tends to diffuse in the form of Li_2_O at high temperatures, and its diffusion rate is much faster than that of transition metal elements. Moreover, the 3D lithium-ion conductive pathway in spinel-type LTO and the high lithium-ion mobility in NASICON-type LATP also provide convenient channels for the diffusion of Li element. Based on crystallographic calculations [47,48], the theoretical Li concentration in the Li_1.3_Al_0.3_Ti_1.7_(PO_4_)_3_ structure is about 9.94 × 10^−3^ mol/cm^3^, much lower than the 29.8 × 10^−3^ mol/cm^3^ in Li_4_Ti_5_O_12_. Consequently, the reaction between LATP and LTO during co-sintering is mainly caused by the diffusion of Li from LTO into LATP. On the one hand, the loss of Li from LTO leads to its decomposition into the TiO_2_, while on the other hand, LATP decomposes due to the excess Li_2_O, forming Li_3_PO_4_ and LiTiOPO_4_, possibly along with a small amount of AlPO_4_ and TiO_2_.

**Figure 7:**
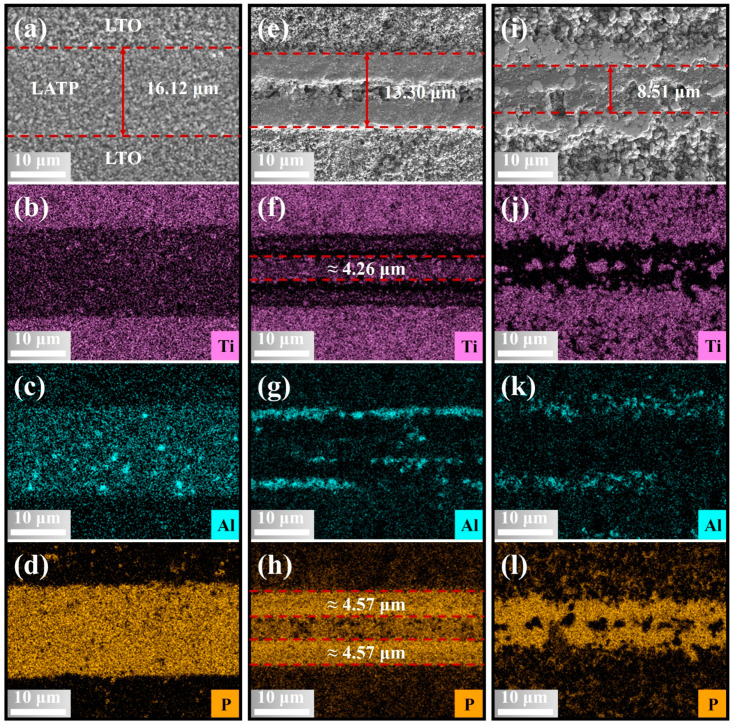
FESEM-EDS mapping of LATP-LTO multilayer composite sheets: (**a**–**d**) green sheets, (**e**–**h**) co-sintered at 800 °C, and (**i**–**l**) co-sintered at 900 °C.

To further investigate the multilayer co-sintering characteristics of LATP and LTO, the LATP-LTO multilayer composite sheets were fabricated. Figure 7 shows the morphology and EDS mapping of PT sheets before and after co-sintering. In general, LATP and LTO can be co-sintered together well at 800–900 °C, with relatively tight interlayer bonding and no significant warping or delamination, thereby meeting the basic conditions for the successful fabrication of MLCBs.

Compared to the PT green sheets, the LATP layer in the PT sheets co-sintered at 800 °C exhibits good densification on both sides. The thickness of the LATP layer decreases to approximately 13.3 µm, with the dense regions near the LATP/LTO interfaces measuring about 4.57 µm on each side, as showed in Figure 7e,h. And a non-densified layer of approximately 4.26 µm remains in the middle, as showed in Figure 7f. However, when the co-sintering temperature reaches 900 °C, the LATP layer becomes almost fully densified, with its thickness reduced to 8.51 µm, as shown in Figure 7i. Meanwhile, the isolated light gray particles appear in the densified central region, and the LTO layer near the LATP/LTO interface also becomes highly densified. The EDS mapping in Figure 7, along with the EDS point analysis of Area 3 in Figure S5a and Spot 2 in Figure S5b, collectively demonstrates that the densified dark gray regions in the LATP layer are predominantly composed of P and O, with relatively low contents of Ti and Al. The isolated light gray particles in the middle region of the LATP layer and the light gray region near the LATP/LTO interface have a close elemental composition, primarily consisting of Ti and O. Based on the previous analysis of the phase structure and morphologies evolution of PT pellets, it is evident that the densified dark gray regions in the LATP layer are primarily Li_3_PO_4_, while the light gray particles in the middle region and the light gray regions near the LATP/LTO interface are primarily TiO_2_. Moreover, after co-sintering the PT sheets, it is observed that the Al element predominantly concentrates near the LATP/LTO interface, likely due to the precipitation of the Al-containing amorphous liquid phase along the interface during the cooling stage.

Based on the above analysis, we can summarize the co-sintering interface reaction mechanism of the LATP-LTO sheets. As the co-sintering temperature increases, Li_2_O in the LTO layer gradually diffuses along the LATP/LTO interface and penetrates the entire LATP layer, inducing the in situ decomposition of LATP inward along the interface to form Li_3_PO_4_. This process promotes the sintering densification of the LATP layer and the LTO layer near the LATP/LTO interface. Correspondingly, the LTO layer decomposes into TiO_2_ due to the loss of Li_2_O. Additionally, comparing the EDS point analysis results of Area 1 between Figure S5a,b, it is observed that the Ti content in the LTO layer increases and the O content decreases after co-sintering at 900 °C compared to 800 °C, providing evidence of Li_2_O loss in the LTO layer. It is worth noting that, for the co-sintering of multilayer heterogeneous materials, a moderate interface reaction is crucial for improving interface contact and enhancing interface bonding strength. Encouragingly, Li_3_PO_4_ serves this function and preserves some ionic conductivity, while TiO_2_ is also a promising anode material [49,50]. These properties are beneficial for the fabrication of MLCBs.

## 4. Conclusions

This study systematically reveals the chemical compatibility between the LATP solid-state electrolyte and the LTO anode during co-sintering, by investigating the thermal behavior, phase structure, morphological evolution, and elemental chemical states of LATP-LTO composite electrodes co-sintered at different temperatures. The results indicate that during the co-sintering process, the diffusion of Li from LTO into LATP leads to the in situ decomposition of LATP and LTO, without the release of oxygen or changes in elemental valence states. The final products are primarily composed of TiO_2_, Li_3_PO_4_, LiTiOPO_4_, and Al-containing amorphous compounds. Moreover, the multilayer co-sintering characteristics of LATP and LTO were further investigated by constructing LATP-LTO multilayer composite sheets. It was found that LATP and LTO can be co-sintered together well at 800–900 °C, primarily due to the formation of Li_3_PO_4_ near the LATP/LTO interface. During the co-sintering process, Li_3_PO_4_ not only promotes sintering densification, but also significantly enhances the interfacial bonding between the LATP and LTO layers. These results provide guidance for the application of LATP solid-state electrolytes and LTO anodes in MLCBs. Future research will focus on controlling the diffusion of Li during co-sintering, which may be achieved by optimizing the sintering process, designing interface blocking layers, and employing rapid sintering techniques such as Spark Plasma Sintering (SPS), Thermal Pulse Sintering (TPS), and Microwave Sintering (MS). In addition, the co-sintering of positive and negative electrodes with electrolyte layers together through multilayer ceramic technology like the MLCC process still requires further research. The possible problems such as warping, cracking, and swelling during MLCB co-sintering need to be solved by adjusting sintering conditions and additives.

## Data Availability

The original contributions presented in this study are included in the article/Supplementary Materials. Further inquiries can be directed to the corresponding authors.

## Supplementary Materials

The following supporting information can be downloaded at https://www.mdpi.com/article/10.3390/ma18040851/s1: Figure S1: XRD patterns of LATP pellets sintered at different temperatures; Figure S2: XRD patterns of LTO pellets sintered at different temperatures; Figure S3: FESEM image of (a) LATP, (b) LTO, and (c) the cross-sectional of LATP-LTO composite green pellet; Figure S4: (a) TEM image of LATP-LTO composite pellet co-sintered at 800 °C; (b–d) HRTEM images corresponding to the red, green, and blue squares in (a), respectively; Figure S5: FESEM-EDS spot/area analysis of LATP-LTO tapes co-sintered at (a) 800 °C and (b) 900 °C.
