# Supplementary material for: Chemical Compatibility of Li1.3Al0.3Ti1.7(PO4)3 Solid-State Electrolyte Co-Sintered with Li4Ti5O12 Anode for Multilayer Ceramic Lithium Batteries
Source: Materials (Basel). 2025 Feb 15;18(4):851. doi: 10.3390/ma18040851 (PMC11857131; doi:10.3390/ma18040851)
Supplement: Supplementary file 1 [file materials-18-00851-s001.zip › materials-3475372-supplementary.pdf]

materials

Article

Chemical Compatibility of $Li_{1.3}Al_{0.3}Ti_{1.7}(PO_4)_3$ Solid-State Electrolyte Co-Sintered with $Li_4Ti_5O_{12}$ Anode for Multilayer Ceramic Lithium Batteries

Jiangtao Li [1,2], Mingsheng Ma [1,2,*], Ya Mao [3], Faqiang Zhang [1], Jingjing Feng [1], Yingchun Lyu [4], Tu Lan [4], Yongxiang Li [5], and Zhifu Liu [1,2,*]

[1] State Key Laboratory of High Performance Ceramics and Superfine Microstructure, Shanghai Institute of Ceramics, Chinese Academy of Sciences, Shanghai 201899, China; lijiangtao22@mails.ucas.ac.cn (J.L.)
[2] Center of Materials Science and Optoelectronics Engineering, University of Chinese Academy of Sciences, Beijing 100049, China; lijiangtao22@mails.ucas.ac.cn (J.L.)
[3] State Key Laboratory of Space Power Sources, Shanghai Institute of Space Power-Sources, Shanghai 200245, China
[4] College of Sciences and Institute for Sustainable Energy, Shanghai University, Shanghai 200444, China
[5] School of Engineering, RMIT University, Melbourne 3000, Australia
* Correspondence: mamingsheng@mail.sic.ac.cn (M.M.); liuzf@mail.sic.ac.cn (Z.L.)

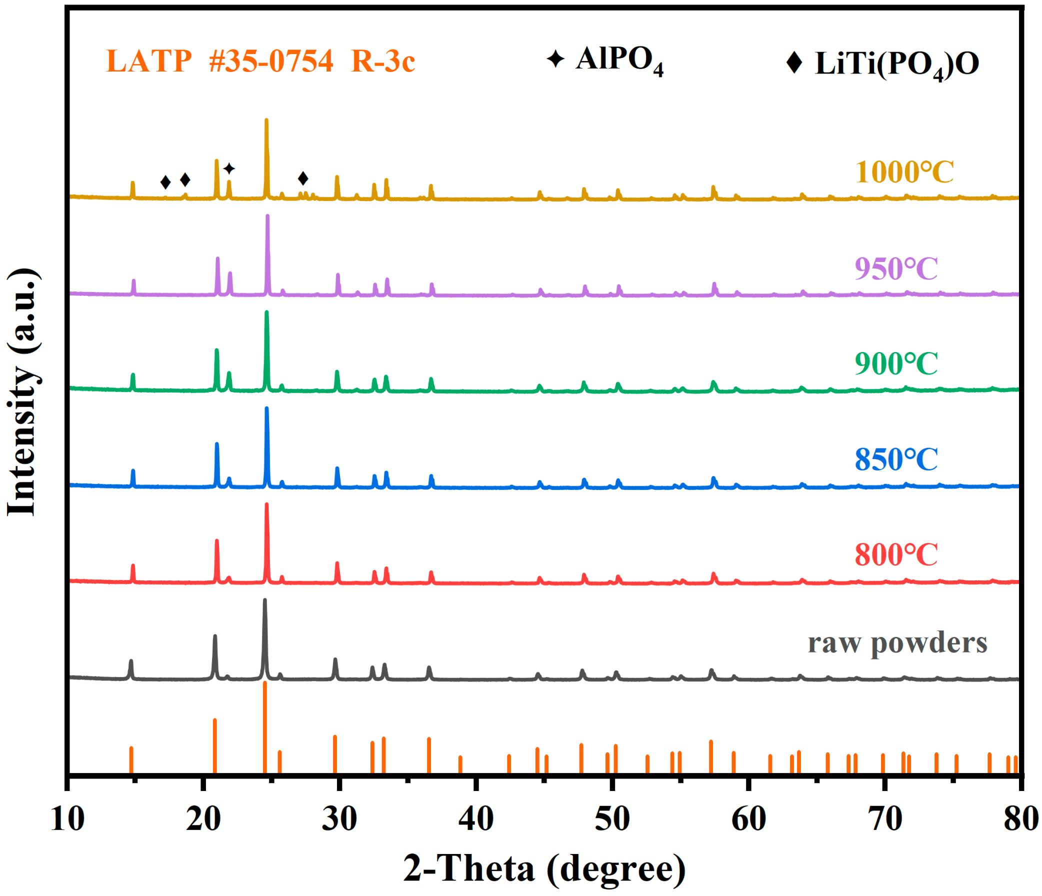

Figure S1. XRD patterns of LATP pellets sintered at different temperatures.

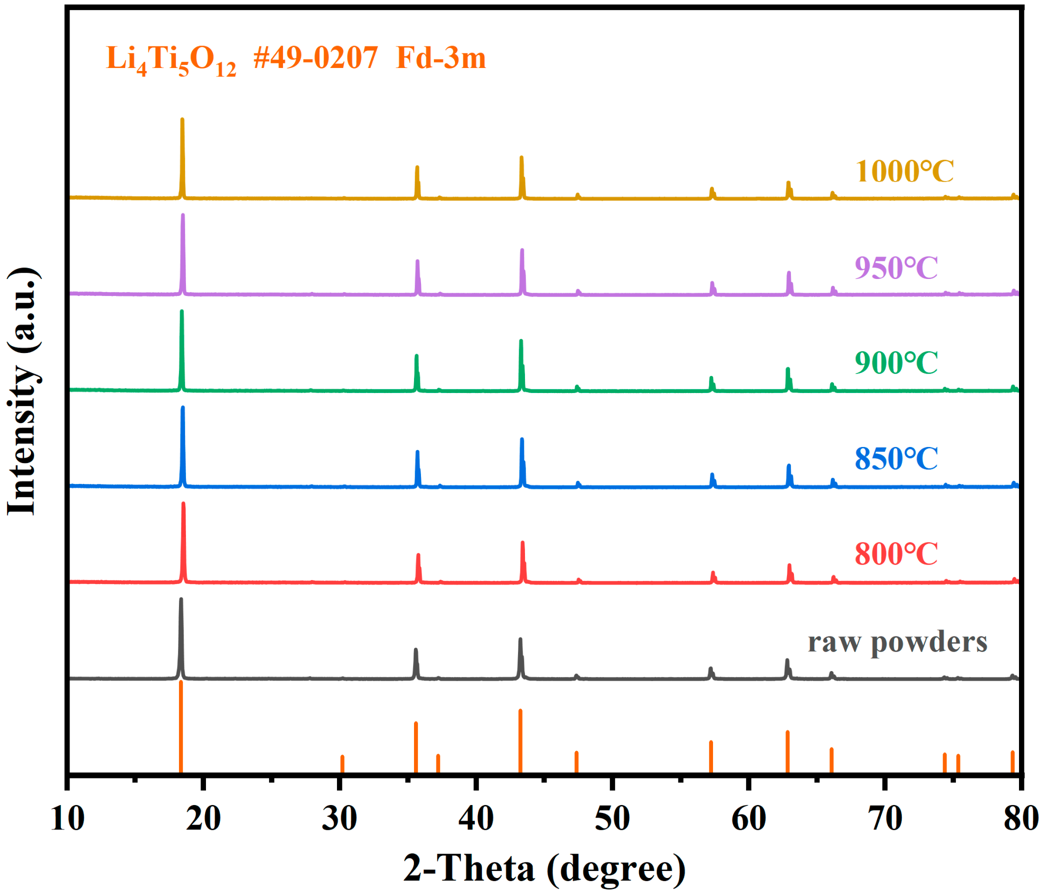

Figure S2. XRD patterns of LTO pellets sintered at different temperatures.

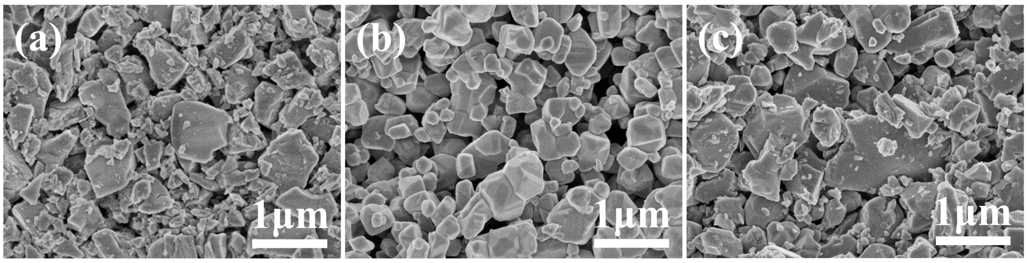

Figure S3. FESEM image of (a) LATP, (b) LTO and (c) the cross-sectional of LATP-LTO composite green pellet.

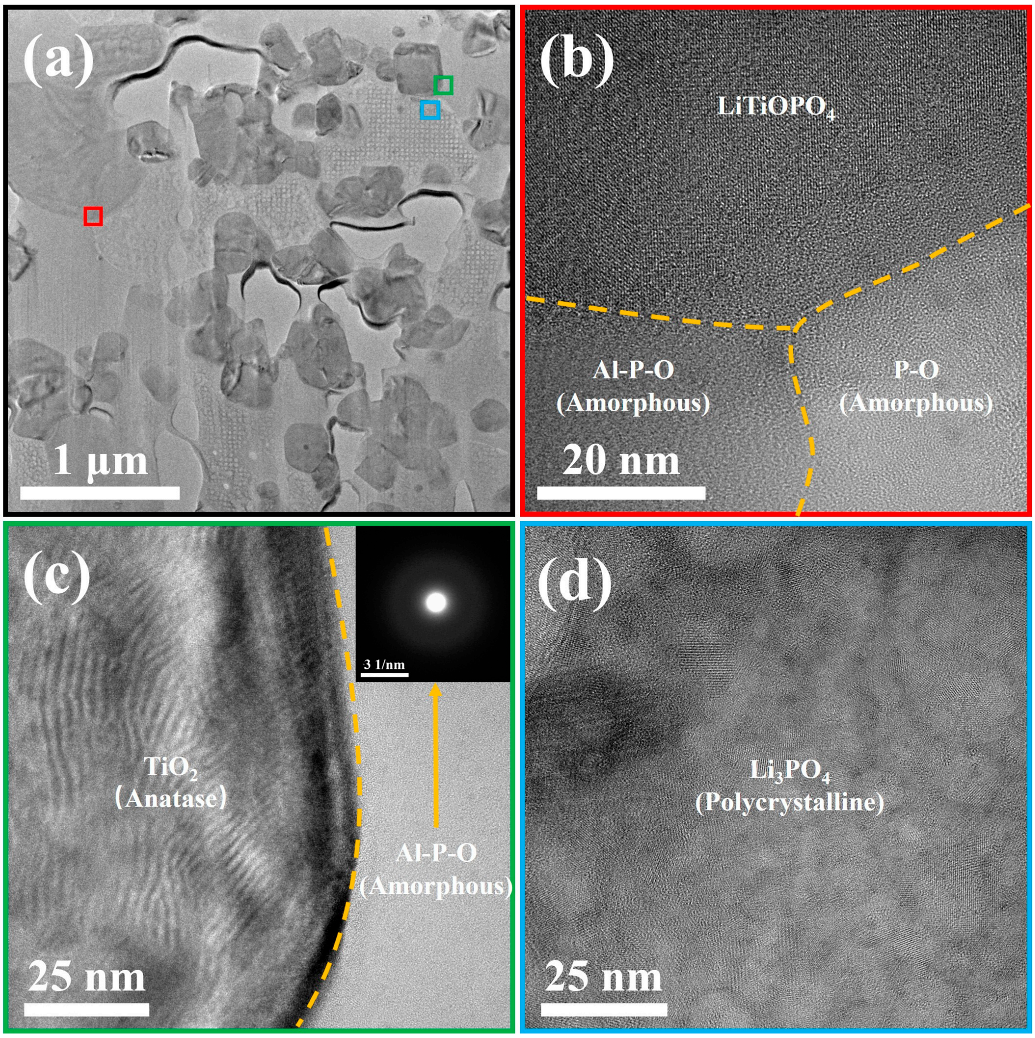

Figure S4. (a) TEM image of LATP-LTO composite pellet co-sintered at 800 °C; (b-d) HRTEM images corresponding to the red, green, and blue squares in (a), respectively.

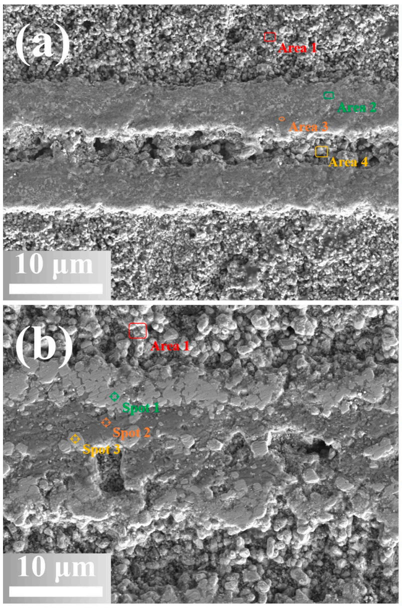

element	Area 1	Area 2	Area 3	Area 4
Ti	26.77%	7.83%	3.10%	14.31%
Al	0.20%	0.40%	0.20%	0.40%
P	2.32%	13.86%	21.30%	11.39%
O	70.71%	77.91%	75.40%	73.89%

element	Area 1	Spot 1	Spot 2	Spot 3
Ti	40.47%	29.70%	1.40%	28.47%
Al	0.00%	0.40%	0.00%	0.20%
P	3.06%	1.30%	24.00%	1.60%
O	56.47%	68.60%	74.60%	69.73%

Figure S5. FESEM-EDS spot/area analysis of LATP-LTO tapes co-sintered at (a) 800 °C and (b) 900 °C.
